# Studies of modern Italian dog populations reveal multiple patterns for domestic breed evolution

**DOI:** 10.1002/ece3.3842

**Published:** 2018-02-14

**Authors:** Andrea Talenti, Dayna L. Dreger, Stefano Frattini, Michele Polli, Stefano Marelli, Alexander C. Harris, Luigi Liotta, Raffaella Cocco, Andrew N. Hogan, Daniele Bigi, Romolo Caniglia, Heidi G. Parker, Giulio Pagnacco, Elaine A. Ostrander, Paola Crepaldi

**Affiliations:** ^1^ Dipartimento di Medicina Veterinaria Università di Milano Milano Italy; ^2^ National Human Genome Research Institute National Institutes of Health Bethesda MD USA; ^3^ Dipartimento di Scienze Veterinarie University of Messina Messina Italy; ^4^ Dipartimento di Medicina Veterinaria University of Sassari Sassari Italy; ^5^ Dipartimento di Scienza e Tecnologie Agro‐Alimentari Alma Mater Studiorum University of Bologna Bologna Italy; ^6^ Area per la Genetica della Conservazione Istituto Superiore per la Protezione e la Ricerca Ambientale Ozzano dell'Emilia Bologna Italy

**Keywords:** canine, domestication, haplotypes, Italian, SNP

## Abstract

Through thousands of years of breeding and strong human selection, the dog (*Canis lupus familiaris*) exists today within hundreds of closed populations throughout the world, each with defined phenotypes. A singular geographic region with broad diversity in dog breeds presents an interesting opportunity to observe potential mechanisms of breed formation. Italy claims 14 internationally recognized dog breeds, with numerous additional local varieties. To determine the relationship among Italian dog populations, we integrated genetic data from 263 dogs representing 23 closed dog populations from Italy, seven Apennine gray wolves, and an established dataset of 161 globally recognized dog breeds, applying multiple genetic methods to characterize the modes by which breeds are formed within a single geographic region. Our consideration of each of five genetic analyses reveals a series of development events that mirror historical modes of breed formation, but with variations unique to the codevelopment of early dog and human populations. Using 142,840 genome‐wide SNPs and a dataset of 1,609 canines, representing 182 breeds and 16 wild canids, we identified breed development routes for the Italian breeds that included divergence from common populations for a specific purpose, admixture of regional stock with that from other regions, and isolated selection of local stock with specific attributes.

## 1 INTRODUCTION

Arising from wild gray wolves on the Eurasian continent over 15,000 years ago, the dog (*Canis lupus familiaris*) was the first species to be domesticated (Frantz et al., 2016; Freedman et al., 2014; Nobis, 1979; Savolainen, Zhang, Luo, Lundeberg, & Leitner, 2002). Mitochondrial DNA evidence suggests the seat of canine domestication was either China (Savolainen et al., 2002), Europe (Thalmann et al., 2013), or the Middle East (von Holdt et al., 2010). Since domestication, the species has undergone thousands of years of selective breeding, giving rise to a myriad of phenotypic variants. However, most modern breeds are 200 years old and are of European ancestry (Club, 2006, 2017; Lindblad‐Toh et al., 2005; Parker et al., 2017).

High‐density, genome‐wide breed phylogeny analysis has been applied to well‐established dog breeds to elucidate the complex structure of breed relationships and their development (Dreger, Davis, et al., 2016; von Holdt et al., 2010; Mortlock, Khatkar, & Williamson, 2016). The largest phylogenetic study reported to date includes 161 dog breeds and over 1,300 dogs, consisting of 23 cladistic groupings based on genetic similarities (Parker et al., 2017). Smaller studies exist which have addressed population demography in globally recognized breeds sampled from discrete locations with some success (Bigi, Marelli, Randi, & Polli, 2015; Ceh & Dovc, 2014; Dreger, Davis, et al., 2016; Koskinen & Bredbacka, 2000; Oliehoek, Bijma, & van der Meijden, 2009; Parra, Mendez, Canon, & Dunner, 2008; Pertoldi et al., 2013; Pribanova et al., 2009; Suarez, Betancor, Fregel, & Pestano, 2013; Wiener et al., 2017). While such studies provide insight into the status and genetic health of local populations, they do not necessarily consider the preexisting breed‐specific genomic patterns developed prior to localization or the impact of import and export from the local breeding pool. Additionally, most prior attempts to address the genetic history and composition of so called “niche” dog populations have used low depth genome coverage incorporating only SNPs, microsatellites, or mitochondrial DNA, and utilizing a small number of breeds which are frequently chosen for their modern population numbers (Ceh & Dovc, 2014; Kang et al., 2009; Pires et al., 2006; Puja, Irion, Schaffer, & Pedersen, 2005; Suarez et al., 2013). These approaches address only superficial relatedness, diminishing the impact of artificial selection, natural divergence, and directed hybridization of breeds, which are evident only through analysis of deep genetic data.

Purebred dog registries, such as the American Kennel Club (AKC) or the Fédération Cynologique Internationale (FCI), attempt to classify dog populations as distinct breeds by enforcing regulations of pedigree tracking, adherence to a written standard, and population size. A genomic pattern for breed status classification based on values from 79 globally recognized purebreeds has recently been defined and includes threshold levels from four homozygosity measurements (Dreger, Davis, et al., 2016; Dreger, Rimbault, et al., 2016). These metrics demarcate the extremes of single‐dog length of homozygosity, ten‐dog shared length of homozygosity, coefficient of inbreeding, and rate of shared homozygosity decay represented by standardized pure dog breeds.

The Italian Kennel Club (ENCI) was founded in 1882 with the registration of the Bracco Italiano pedigree (ENCI). Today, ENCI recognizes 16 different Italian breeds (ENCI), of which 13 are utilized in this study. Additionally, seven local landrace populations, defined as distinct dog varieties unique to a specific geographic region with historically limited breeding populations, are included in this study. The latter are not recognized by any purebred canine registry but, nonetheless, may display a genetic pattern consistent with other purebreeds (Alam, Han, Lee, Ha, & Kim, 2012; Puja et al., 2005; Tanabe, 2007; Wijnrocx, Francois, Stinckens, Janssens, & Buys, 2016; Yoo et al., 2017), such as has been observed for one Italian regional population, the Fonni's Dog (Dreger, Davis, et al., 2016; Dreger, Rimbault, et al., 2016; Sechi et al., 2016). By focusing our analyses on breeds with diverse phenotypes that have all originated in a single country, we aim to employ genetic data to expand upon historical breed formation accounts and define the modes by which humans have produced recognizable and diversified dog breeds. While smaller‐scale studies have identified genetic routes of breed development relative to discrete dog breed types (Akkad, Gerding, Gasser, & Epplen, 2015; Parra et al., 2008), these aims have not yet been applied to a large, comprehensive representation of diverse breeds.

**Figure 1:**
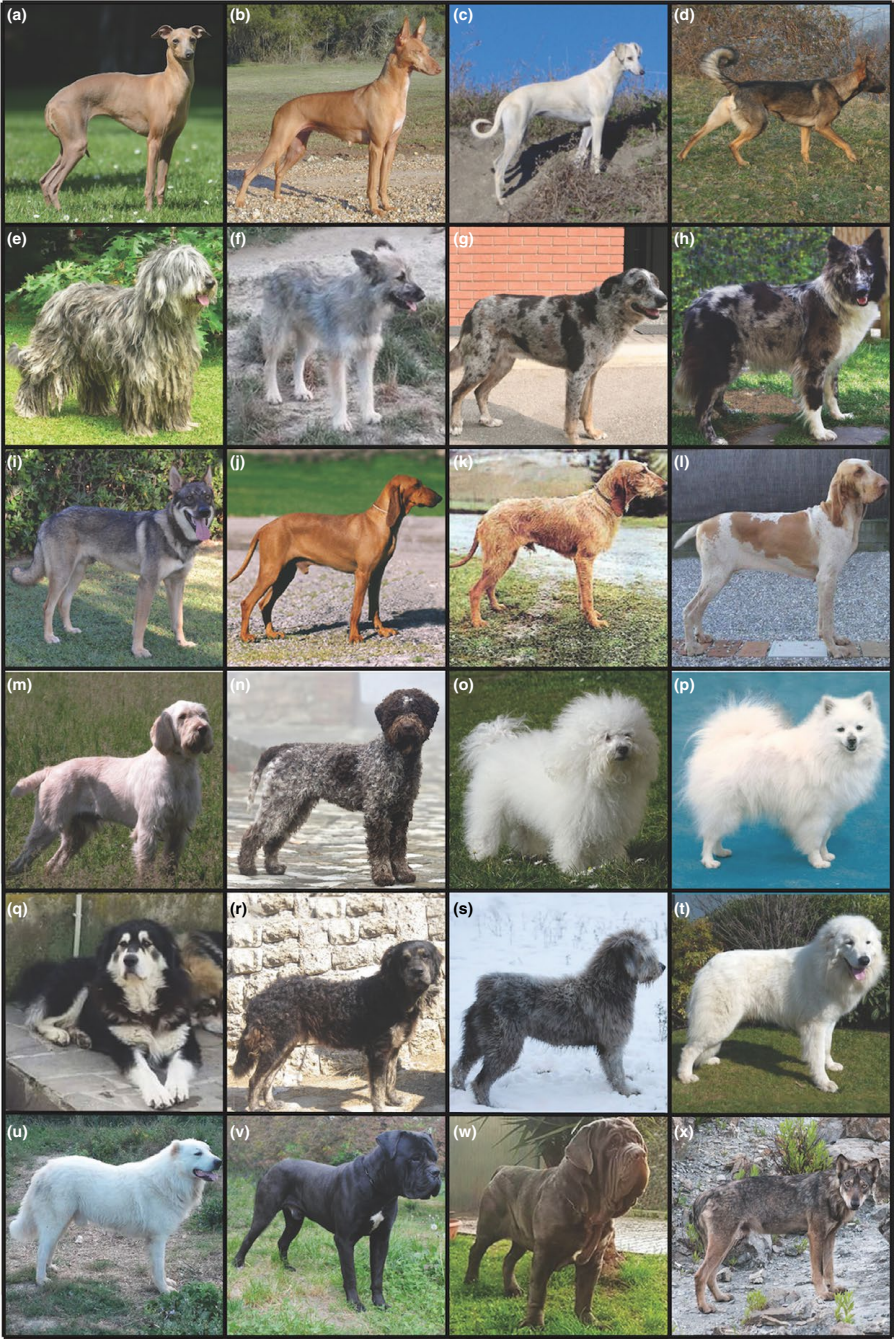
Italian breeds used to model modes of breed development. Sighthounds: (a) Italian Greyhound, (b) Cirneco dell'Etna, and (c) Levriero Meridionale. Wolf hybrid: (d) Lupo Italiano. Herding breeds: (e) Bergamasco Shepherd, (f) Cane Paratore, (g) Pastore d'Oropa, (h) Pastore della Lessinia e del Lagorai, and (i) Lupino del Gigante. Scent hounds: (j) Segugio Italiano Pelo Raso and (k) Segugio Italiano Pelo Forte. Hunting breeds: (l) Bracco Italiano, (m) Spinone Italiano, and (n) Lagotto Romagnolo. Companion breeds: (o) Bolognese and (p) Volpino Italiano. Livestock guardian breeds: (q) Pastore della Sila, (r) Mannara's Dog, (s) Fonni's Dog, (t) Maremma Sheepdog, and (u) Mastino Abruzzese. Mastiffs: (v) Cane Corso and (w) Neapolitan Mastiff. Wild canid: (x) Apennine wolf. Photograph credits are listed in the Acknowledgements

In this study, we investigated 23 dog populations of Italian origin (Table 1, Figure 1) and a sampling of seven wild wolves belonging to the Italian gray wolf population which were collected from the Italian Apennine mountain ranges (hereafter referred to as “Apennine wolves”; Table 1). The Italian breeds were derived from populations selected for hunting, tracking, herding, property and livestock guarding, coursing, and companionship, each having a long and distinct history of development in niche Italian regions. We have determined the genetic population structure of these regional populations relative to a large sampling of 161 global dog breeds with well‐established relationships (Parker et al., 2017) using data from the ~170K Illumina CanineHD SNP array. With a total of 1,609 domestic dogs, representing 182 breeds, and 16 wild canids, we have assembled the largest and most diverse dataset of canine genomes to determine breed status of domestic dog varieties in a singular geographic region. Utilizing analyses of phylogeny, identity‐by‐descent haplotype sharing, and admixture, we identify specific instances of three modes by which dog breeds have been formed: (1) specialization of breeds through segmentation within a phenotypically similar population; (2) directed attainment of common species‐wide phenotypes within multiple diverse regionally isolated gene pools; (3) introduction of desired characteristics through introgression with distantly related populations.

**Table 1:** The phenotypic classification and level of registration of the twenty‐three Italian dog populations under investigation

Breed	Type^a^	Registry^b^
Bergamasco Shepherd	Herding	ENCI, AKC
Bolognese	Companion	ENCI, FSS
Bracco Italiano	Pointer	ENCI, FSS
Cane Corso	Mastiff	ENCI, AKC
Cirneco dell'Etna	Sighthound	ENCI, AKC
Cane Paratore	Herding	Local to Abruzzo
Fonni's dog	Livestock guardian	Local to Sardinia
Italian greyhound	Companion	ENCI, AKC
Lagotto Romagnolo	Water dog	ENCI, AKC
Levriero Meridionale	Sighthound	Local to Southern Italy
Lupino del Gigante	Herding	Local to Emilia‐Romagna
Lupo Italiano^c^	Apennine wolf hybrid	Nationally managed
Mannara's dog	Livestock guardian	Local to Sicily
Maremma sheepdog	Livestock guardian	ENCI
Mastino Abruzzese	Livestock guardian	Local to Abruzzo
Neapolitan Mastiff	Mastiff	ENCI, AKC
Pastore della Lessinia e del Lagorai	Herding	Local to Northeast Italy
Pastore della Sila	Livestock guardian	Local to Calabria
Pastore d'Oropa	Herding	Local to Lombardy
Segugio Italiano Pelo Forte	Scent hound	ENCI
Segugio Italiano Pelo Raso	Scent hound	ENCI
Spinone Italiano	Pointer	ENCI, AKC
Volpino Italiano	Companion	ENCI
Apennine wolf	Wild canid	Apennines and Western Alps

a Type based on physical and behavioral characteristics.

b If nationally recognized, the breed registry is listed as Italian Kennel Club (ENCI), American Kennel Club (AKC), or AKC foundation stock service (FSS). If not nationally recognized, the Italian region of popularity is indicated.

c The Lupo Italiano was reportedly produced through breeding of an Apennine wolf and multiple German Shepherd Dogs. The breed is closely managed and maintained by the Italian government.

## 2 MATERIALS AND METHODS

### 2.1 Samples

Blood samples from 256 dogs and seven Apennine wolves (Table 1) were collected following the European Rules for Animal Welfare when collected in Italy and approved National Human Genome Research Institute (NHGRI) Animal Care and Use Committee protocols when collected or received in the United States. The dog populations represent 23 breeds or varieties of historical Italian origin. The ENCI recognizes 13 of these populations as purebreeds and nine of which are also recognized by the AKC. Ten populations are termed “varieties,” and represent regional homogeneous populations, generally managed by informal registries and maintained by owners for specific behavioral applications. Three breeds, the Cane Corso, Italian Greyhound, and Neapolitan Mastiff, were sampled from Italian populations to compliment a preexisting collection of American populations of the same breeds. Animals selected for analysis were as distantly related as possible based on pedigree information from ENCI. DNA extraction was performed with the commercial Qiagen DNeasy Blood & Tissue Kit. Samples were genotyped on the Illumina CanineHD bead chip (Illumina, San Diego, CA, USA), which contains 172,115 potential markers, in the Ostrander laboratory at the National Human Genome Research Institute (NHGRI) of the National Institutes of Health (Bethesda, MD, USA) using manufacturer's recommended protocols.

Genotyped samples were merged with a larger dataset of 1,346 dogs representing 161 breeds (described in (Parker et al., 2017)) and publically available genotypes from five New Guinea Singing Dogs and three Catahoula Leopard Dogs (Hayward et al., 2016) to produce a dataset of 1,609 dogs representing 182 breeds, seven Apennine wolves, seven global Grey wolf representatives, and two Golden Jackals.

### 2.2 Data editing

The initial genotype dataset was screened to remove all SNPs with a call rate < 95% and a minor allele frequency<0.01% using Plink 1.9 software (Purcell et al., 2007). Only markers on autosomes were retained for subsequent analysis, resulting in 142,840 SNP variants. After low‐quality marker exclusion, dogs with a low call rate (<90%) were excluded. Duplicated individuals were detected using identity‐by‐state (IBS) with a cutoff of >99%. One animal for each identical pair was excluded from subsequent analysis. Closely related pairs of dogs were identified and pruned using discordant homozygote count (Mendelian error, ME), where a pair of individuals was classified as related if their ME was <100.

### 2.3 Phylogenetic and genetic distances estimation

**Figure 2:**
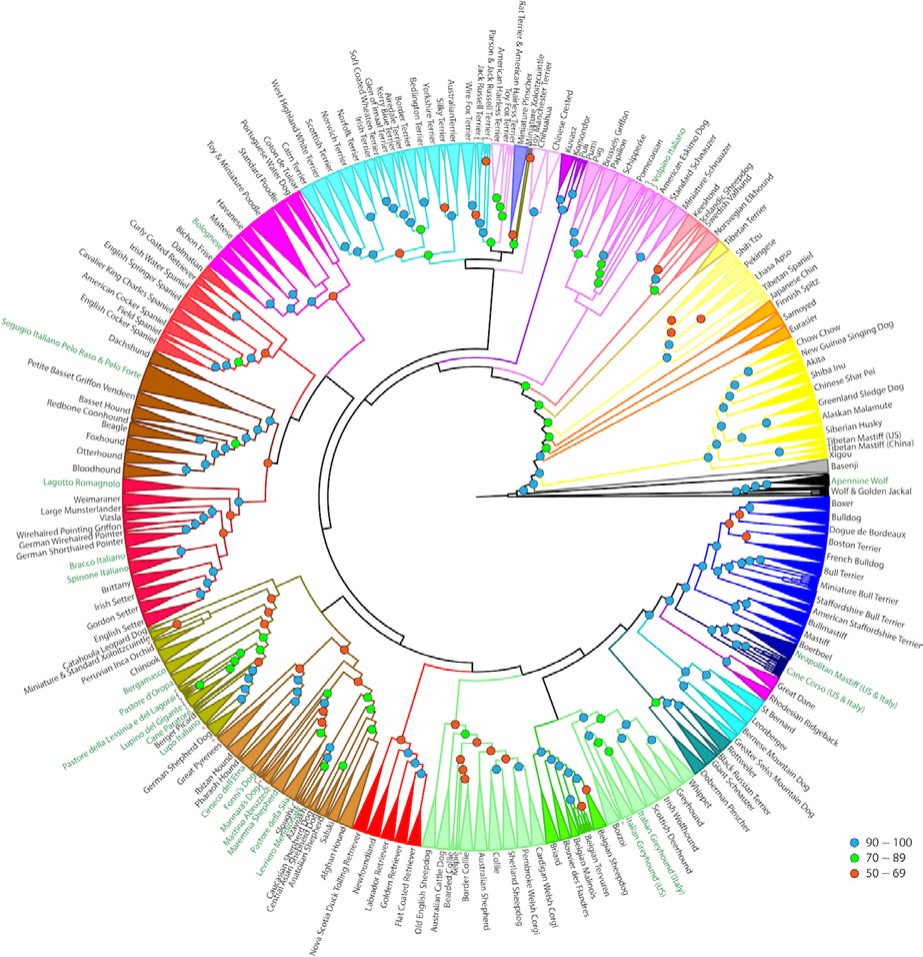
Phylogeny dendrogram calculated by genetic distance. The phylogeny was bootstrapped 100 times, nodes greater than 50% confidence are indicated. All breeds are clustered with 100% confidence unless otherwise indicated. Clades are colored relative to the genetic distance relationships. Italian breeds are highlighted with green text

All breeds in the dataset were resized to a maximum of 10 randomly selected individuals to avoid size‐related bias affecting phylogenetic analysis. For individuals belonging to Cane Corso, Neapolitan Mastiff, and Italian Greyhound breeds, a maximum of 10 individuals were allowed for both sampling locations. Genetic distances between individuals were estimated using the “—distance” function of Plink 1.9 (Purcell et al., 2007) and the “1‐ibs,” “square,” and “flat‐missing” modifiers, with 100 bootstraps. Neighbor‐joining phylogeny and consensus tree calculation, with Golden Jackal as the outgroup, were conducted using the PHYLIP software package (Felsenstein, 1989). Any dogs that did not group according to their expected breed with >90% bootstrapping confidence were considered to be misclassified and removed from subsequent analyses. Breeds were assigned to clades, represented by different colors in Figure 2, relative to the expected organization presented in Parker et al. (Parker et al., 2017).

### 2.4 Haplotype sharing

Haplotype sharing, as determined by identity‐by‐descent estimations among individuals, was calculated using the Beagle v4.1 software (Browning & Browning, 2013). Haplotypes shared between individuals, estimated on all markers, were reconstructed using 100‐SNP sliding windows with a step of 40 markers each and allowing for trimming of up to 10 markers. The sum of shared haplotype lengths for each pair of dogs from different clades was detected, allowing for the calculation of the median length of shared haplotypes between all dogs of each pair of cross‐clade breeds. All individual pairs that had no haplotype sharing were considered to have a median estimation of shared haplotypes of length = 0. Haplotype sharing was considered to be significant when median values were above the 95th percentile (9,257,455 bp) of all cross‐clade breed pairs. The relative age of shared genetic history between breeds was estimated using previously published methods based on median size of haplotype sharing (Parker et al., 2017).

### 2.5 Single clade analysis and selection signature detection

Subsequent analyses on phylogenetic clades containing Italian breeds included complete datasets and were not limited to 10 dogs per breed. Where necessary, clades with breeds showing substantial haplotype sharing with Italian breeds were included in follow‐up analyses. Phylogenetic calculation for introgression events was conducted with Treemix software (Pickrell & Pritchard, 2012). The number of admixing events ranged from 0 (no migration events) to *N*, where *N* is the number of breeds in the analyzed branch. The migration model with the lowest absolute residual error was considered ideal.

Population genetic structure analysis was performed using the fastSTRUCTURE software (Raj, Stephens, & Pritchard, 2014). Population divisions (*K*) range from two to the number of breeds within each cladistic analysis. The optimal *K* value was determined by maximum likelihood of the subdivision (Pritchard, Stephens, & Donnelly, 2000).

### 2.6 Shared homozygosity, decay rate, and inbreeding coefficient calculation

Shared genetic homozygosity (LnH) and homozygosity decay rates for each breed were calculated from the IlluminaHD SNP chip genotypes using Plink 1.9 (Purcell et al., 2007) as outlined previously (Dreger, Davis, et al., 2016; Dreger, Rimbault, et al., 2016). Three breeds were collected in both the United States and Italy and maintained for independent analyses. Breeds were pruned to a maximum of ten random individuals, and only breeds with a minimum of ten individuals were used in LnH calculations. Calculation of decay rate was conducted for all breeds with a minimum of five individuals. All Italian breeds, however, were included in the calculations for single‐dog homozygosity and SNP‐based inbreeding coefficient (*F*). The breed‐specific F was determined by averaging individual *F* values for all dogs of a single breed. These values were compared to previously reported purebred genetic parameters (Dreger, Davis, et al., 2016; Dreger, Rimbault, et al., 2016).

## 3 RESULTS

### 3.1 Breed status classification through genomic metrics

In a previous study, we analyzed a population of 79 dog breeds to outline the genetic parameters of homozygosity and inbreeding expected from purebred populations (Dreger, Rimbault, et al., 2016). These metrics were likewise calculated for the Italian breeds listed in Table 2 and compared to the expected purebred values as follows: single dog LnH ≥ 1098.365 Mb, 10 dog shared LnH ≥ 48.092 Mb, homozygosity decay ≤ 0.607, *F* ≥ 0.133 (Dreger, Davis, et al., 2016; Dreger, Rimbault, et al., 2016. Fifteen of the Italian dog breeds or populations expressed values within purebred ranges for three or more of the four metrics and presented as a single‐breed‐specific clade in the bootstrapped phylogeny. We found that 12 of the initial 23 Italian populations can be classified as “breeds,” including four not yet recognized by ENCI or AKC (Table 2). Seven of the initial 23 populations have sufficient measures of homozygosity and inbreeding to qualify as breeds, but fail to cluster as unique breed‐specific phylogenetic clades and are therefore categorized as paraphyletic “varieties” of other populations. Exceptions of note include the two hair‐type populations of Segugio Italianos which, when treated as one population, qualify as a breed. Also, the Pastore della Sila falls short on measures of breed or variety distinction.

**Table 2:** Genetic metrics of population structure for each Italian dog population

Breed	*N* a	*F* b	1‐Dog LnH (Mb)^c^	Shared LnH (Mb)^d^	Decay^e^	Phylogeny clustering	Status^f^
Bergamasco Shepherd	9	0.220	1843.258	382.935	0.450	Yes	Breed
Bolognese	18	0.147	1848.889	251.647	0.336	Yes	Breed
Bracco Italiano	9	0.139	1893.965	663.608	0.305	Yes	Breed
Cane Corso (Italy)	16	0.139	1803.263	178.954	0.380	No	Variety
Cane Corso (US)	4	0.141	1818.177	—	—	No	
Cirneco dell'Etna	14	0.222	1952.620	418.463	0.241	Yes	Breed
Cane Paratore	2	**0.081** g	1862.197	—	—	No	Insuff
Fonni's dog	6	0.162	1839.483	452.063	**0.629**	Yes	Breed
Italian Greyhound (Italy)	10	0.250	1904.847	551.779	0.286	Yes^h^	Breed^h^
Italian Greyhound (US)	10	0.290	1926.256	607.852	0.192	Yes^h^	
Lagotto Romagnolo	18	0.171	1832.910	378.569	0.298	Yes	Breed
Levriero Meridionale	2	0.220	1928.855	—	—	Yes	Insuff
Lupino del Gigante	10	0.141	1664.250	100.585	0.424	Yes	Breed
Lupo Italiano	24	0.478	1988.448	1400.986	0.280	Yes	Breed
Mannara's Dog	12	**0.114**	1887.253	167.485	0.497	No	Variety
Maremma Sheepdog	14	0.124	1856.195	274.145	0.294	Yes	Breed
Mastino Abruzzese	2	**0.026**	1815.659	—	—	No	Insuff.
Neapolitan Mastiff (Italy)	6	0.318	1897.332	—	0.358	Yes^i^	Breed^i^
Neapolitan Mastiff (US)	6	0.296	1935.719	—	0.292	Yes^i^	
Pastore della Lessinia e del Lagorai	10	**0.079**	1637.732	442.396	0.475	No	Variety
Pastore della Sila	14	**0.092**	1856.336	385.056	**0.665**	Yes	Not
Pastore d'Oropa	15	**0.043**	1807.711	495.420	0.579	Yes	Breed
Segugio Italiano Pelo Forte	16	**0.088**	1835.136	144.898	0.365	Yes^j^	Breed^j^
Segugio Italiano Pelo Raso	16	**0.117**	1880.337	189.692	0.351	Yes^j^	
Spinone Italiano	16	0.136	1870.035	308.590	0.277	Yes	Breed
Volpino Italiano	15	0.213	1912.347	361.279	0.299	No	Variety

a Total number of dogs sampled.

b Length of homozygous regions shared across multiple dogs of a breed, with a minimum of five dogs and a maximum of ten, ≥ 48.092 Mb.

c Sum length of homozygous regions based on SNP chip genotypes, ≥1098.365 Mb.

d Average SNP‐based inbreeding coefficient, ≥ 0.133.

e The rate by which the change in shared homozygosity across individuals of a breed declines with the addition of each same breed dog, ≤0.607.

f Population status is determined as a “breed” if the named population conforms to purebred ranges in at least three of the four genomic metrics and all members group together phylogenetically in the all‐breed analysis. If phylogenetic clustering is absent, in the presence of acceptable purebred metric values, the population is classified as a “variety”. Failure to meet these requirements indicates that the population does not meet the genomic expectations of a purebreed. Such instances are highlighted in bold. Populations analyzed with fewer than five individuals are insufficient to determine a status assignment.

g Values in bold are outside the predefined range for purebred populations.

h The US and Italian populations of Italian Greyhounds qualify as a single breed.

i The US and Italian populations of Neapolitan Mastiff qualify as a single breed.

j The Segugio Italiano Pelo Raso and Pelo Forte qualify as a single breed.

This collection of sample populations allowed visualization of multiple stages of breed formation. While each population is managed by human‐driven selection toward a breed standard, the various populations present distinct time points along the road to breed classification. For the purpose of further analysis, breed classification was considered equivalent for all populations. As such, each “breed,” “variety,” or “nonbreed” was treated as a distinct population in analyses that required prior assignment of identity.

### 3.2 Phylogeny analysis reveals breed formation through convergent and divergent selection

Following sample size reduction to a maximum of ten dogs per breed, 1,609 dogs and 16 wild canids were included in the estimation of genetic distance phylogeny. The final consensus tree of 100 bootstraps included 184 single‐breed clades, each with >90% confidence (Figure 2). The primary clades are generally reflective of those published previously by us (Parker et al., 2017), and breed classifications are consistent with the previous report. However, the phylogenetic placement of the added Italian breeds, and subsequent rearrangement of previously defined cladistic relationships, yielded some surprising results.

First, we observed that two clades, the New World and Mediterranean, significantly rearranged, compared to those previously published (Parker et al., 2017), upon addition of the Italian breeds. All six of the Italian herding breeds were newly assigned to the New World clade. The placement of the German Shepherd Dog is central to our understanding of this group. Other breeds were organized as follows (Figure 2): The Lupo Italiano (Figure 1d) is monophyletic to the German Shepherd Dog. Two herding breeds from North East Italy, the Pastore della Lessinia e del Lagorai (Figure 1h) and Lupino del Gigante (Figure 1i), are monophyletic with each other and are paraphyletic to the Lupo Italiano and German Shepherd Dog. Indicating their incomplete emergence as distinct breeds, a subset of each form two breed‐specific clades with >70% confidence. However, four Pastore della Lessinia e del Lagorai and one Lupino del Gigante fail to fall into either clade, but lie between the two clades, representing a breed development gradient between the two varieties. There appear to be two dogs of the Lupino del Gigante breed and one Pastore della Lessinia e del Lagorai that do not fall near or within the expected clades. These three dogs were likely misclassified at collection, or are resultant of recent hybridization, and are therefore not reflective of their respective breeds. The classification of the Cane Paratore as a breed is highly disputed in Italy. However, the two Cane Paratores (Figure 1f) in this analysis grouped together, paraphyletic to the German Shepherd Dog and Lupo Italiano, suggesting that they are a distinct variety.

The second group undergoing substantial changes was previously termed the “Mediterranean group” as it contained several breeds from the Mediterranean, North African, and Middle Eastern regions (Parker et al., 2017). In this new analysis, the same group now consists of two distinct branches, supported with >50% confidence. This lower confidence likely suggests that the recent genetic history of some breeds links them to both branches. The first branch consists of three sighthounds and a flock guardian: Ibizan Hound, Pharaoh Hound, and Cirneco dell'Etna (Figure 1b) sighthounds, with the Great Pyrenees flock guardian paraphyletic at >90% confidence. The second branch consists of the Italian flock guardians, the Fonni's Dog (Figure 1s), Maremma Sheepdog (Figure 1t), Pastore della Sila (Figure 1q), and Mastino Abruzzese (Figure 1u), and the Italian sighthound, the Levriero Meridionale (Figure 1c), together with flock guardian and sighthound breeds of the Middle East region. The Italian flock guardian breed, Maremma Sheepdog, is central to this branch as the German Shepherd Dog was above. Two Italian flock guardian breeds, the Mannara's Dog (Figure 1r) and the Mastino Abruzzese, branch paraphyletically from the Maremma Sheepdog and Pastore della Sila breed clades, without forming consistent or supported breed‐specific groupings. Some breeds exited the Mediterranean Clade with the addition of the Italian breeds. The Komondor and Kuvasz are now combined with the Pumi and Puli breeds with >90% bootstrapped confidence to expand the “Hungarian” clade.

### 3.3 Haplotype sharing analysis identifies breed specialization through genetic isolation or purposeful admixture

**Figure 3:**
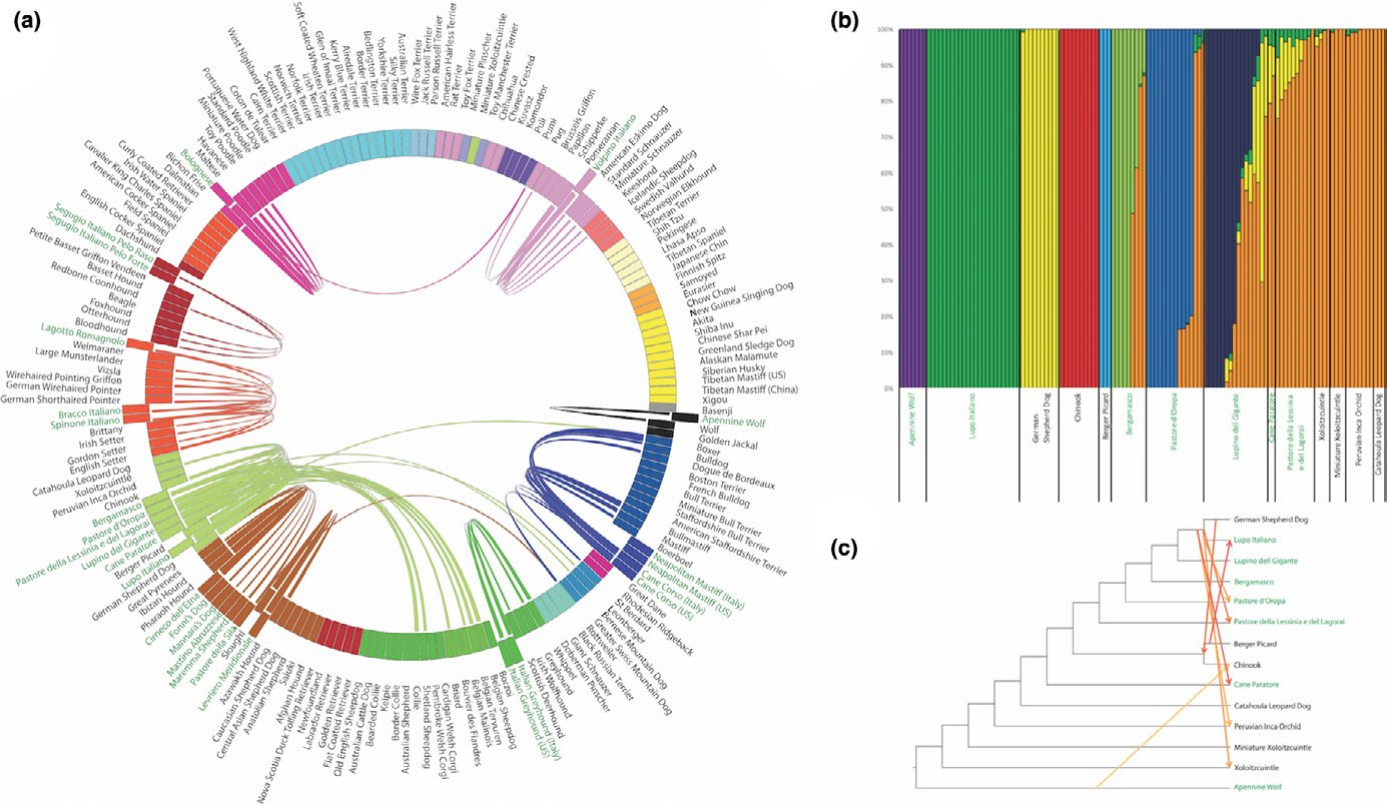
**(**a) Significant haplotype sharing of Italian breeds with non‐Italian breeds. (b) Structure analysis of the New World clade of breeds, plus the Apennine Wolf. *K *=* *10. (c) Treemix prediction of admixture events between breeds from the New World clade, plus the Apennine Wolf. *N* = 8

While phylogenetic analysis displays breed relationships based on sequential differentiation of continually smaller subpopulations, observation of haplotype sharing through identity‐by‐descent accounts for admixture between distantly related breeds. Across‐clade haplotype sharing was calculated for each as determined by the bootstrapped genetic distance phylogeny (Figure 2) to better understand breed development through introgression. Pairwise comparisons of identity‐by‐descent haplotype sharing between individual dogs from different breeds were considered, resulting in a total of 1,240,389 comparisons. The median shared haplotype value of each breed‐to‐breed comparison was then calculated; 287 were >9,257,455bp (top 5% of all haplotype sharing), and these measures were considered significant. While most potential comparisons did not involve an Italian breed, 133 of those observed included one or more Italian breeds (Figure 3, Table S1).

Nine Italian breeds show significant haplotype sharing only with breeds within their own clades (Figure 3, Table S1), indicating breed development through within‐clade divergence. The remaining Italian breeds showed evidence of within‐clade haplotype sharing as well as hybridization with breeds in other clades. This suggests that their breed development relied, in part, on introduction of desirable traits from unrelated breeds. These results describe the specific differences between populations of similar type and function. For instance, three Italian flock guardian breeds show significant haplotype sharing with a fourth Italian flock breed, the Maremma Sheepdog, but not with each other. Rather, they are differentiated by influence of outside breeds: the Pastore della Sila with the Rottweiler and the Mannara's Dog with the German Shepherd Dog. Meanwhile, the fifth Italian flock guardian breed, the Fonni's Dog, does not show haplotype sharing with other breeds that perform the same function, but only has haplotype sharing with the German Shepherd Dog. Overall, this indicates directionality of the introgression used in the formation of these particular breeds. While the directionality speaks to the presence of human intervention, it does not address the issue of whether directed breeding occurred relative to a single or modest number of traits, suggesting purposeful admixture, or more generally to the population dynamics of the region, implicating divergent selection.

Breed haplotype commonality is present within the New World clade, whereby each of the Italian herding breeds has significant haplotype sharing with each of the other Italian herding breeds, as well as the Berger Picard, Chinook, and German Shepherd Dog. Differentiating the Italian herding breeds from each other is varying levels of contribution from 11 additional breeds from the UK Rural, New World, Continental Herder, Alpine, and European Mastiff clades (Table S1). Interestingly, however, for all six of the Italian herding breeds, the German Shepherd Dog haplotype is the most predominant.

To our surprise, we detected several instances where a single Italian herding breed shares genetic history with a breed from outside the clade. For instance, we observed signatures of haplotype sharing between the Bergamasco Shepherd (Figure 1e) and the Briard and Bernese Mountain Dog, the Lupino del Gigante and Leonberger, and the Cane Paratore and Boxer. These patterns of haplotype sharing between breed pairs indicate that each Italian herding population diverged from the others through its individual introgression with outside breeds and that they are not merely geographically separate populations of the same breed. The Lupo Italiano is believed to have been developed from a purposeful cross of a German Shepherd Dog and an Apennine wolf (Figure 1x) in the mid‐1960s. While our data support the strong relationship between the Lupo Italiano and German Shepherd Dog, there is no significant level of haplotype sharing between it and the Apennine wolf.

### 3.4 Single clade analysis

Admixture analysis with TREEMIX and structural analysis with fastSTRUCTURE were conducted on each breed group that contains an Italian breed as a separate test of breed relationships. The ideal breed relationship phylogeny was selected based on the number of admixture events (*N*) that yielded the lowest residual standard error. Likewise, the ideal structural division (*K*) was that which resulted in the greatest likelihood. In the case of the New World clade, the Apennine wolf population was included due to the presumed hybridization history with the German Shepherd Dog to produce the Lupo Italiano.

The patterns of relatedness shown in the neighbor‐joining phylogeny and haplotype sharing (Figure 2) are closely mirrored by TREEMIX and fastSTRUCTURE analysis. Figure 3 presents visualization of population structure for the New World clade, while all other clades that include Italian breeds are featured in the Supplementary Material (Supplementary Figure 1). At *K *=* *10, fastSTRUCTURE analysis identifies four Italian herding breeds from the New World group as having unique genetic signatures: Lupo Italiano, Bergamasco, Pastore d'Oropa (Figure 1g), and Lupino del Gigante (Figure 3). A shared signature is present at high levels in all Cane Paratore, Pastore della Lessinia e del Lagorai, and the non‐Italian Standard and Miniature Xoloitzcuintle, Peruvian Inca Orchid, and Catahoula Leopard Dog. Interestingly, the shared signature is also present in approximately half of the Bergamasco, Pastore d'Oropa, and Lupino del Gigante dogs, suggesting that those breeds demonstrate less cohesion than most well‐established breeds. Admixture analysis of the New World breed group again indicates a substantial level of breed introgression, primarily in the direction from the German Shepherd Dog and the Lupo Italiano outward to the other related breeds (*N* = 8).

Like the New World breed group, the Mediterranean breed group is similarly complex (Supplemental Material). An underlying genetic signature is present in dogs from several regions including the Saluki from the Middle East, the Central Asian Shepherd Dog, the Anatolian Shepherd from Turkey, and Italian dogs, and to a lesser extent, the Great Pyrenees from France, Caucasian Shepherd from Western Asia, and Pharaoh Hound from Malta (*K *=* *10). Among the livestock flock guardian breeds, several have a unique genetic signature. However, only five of the 12 Mannara's Dogs and eight of the 14 Pastore della Silas express the breed‐specific signatures at a level of 50%. This implies that these breeds are comprised of dogs that vary from a central breed definition and that the long history of strong selection which defines many established breeds is lacking in these populations.

### 3.5 Impact of geographic separation on breed identity and differential selection

We additionally examined a set of three breeds for which we collected samples in both the United States and Italy: Italian Greyhounds (Figure 1a), Cane Corsos (Figure 1v), and Neapolitan Mastiffs (Figure 1w). As population substructure has previously been observed in international populations of breeds (Pedersen, Liu, Leonard, & Griffioen, 2015; Pedersen, Liu, McLaughlin, & Sacks, 2012; Quignon et al., 2007), we hope to characterize genetic divergence between US and Italian populations of Italian breeds, caused by importation bottlenecks, regional popular sire effects (Leroy & Baumung, 2010; Pribanova et al., 2009), or variation in selection. All three were correctly grouped by breed. Within the breeds, however, distinctions emerged. The Italian Greyhounds split into two sub‐branches of American and Italian dogs with 90% confidence. The Neapolitan Mastiffs, while forming a distinct breed, did not show separation by country. The Cane Corsos did not group as a breed, rather they are paraphyletic to the Neapolitan Mastiffs, as was noted previously (Parker et al., 2017).

Haplotype sharing analysis was then applied to detect the presence of variable genetic histories between the US and Italian collections, revealing new subtleties (Table S1). First, we found that the American population of the Cane Corso shows significant haplotype sharing with the Rottweiler, reproducing previous results (Parker et al., 2017). Second, the American population of Italian Greyhounds shows significant levels of haplotype sharing with the Toy Manchester Terrier. Finally, no new findings were identified for the Neapolitan mastiffs indicating that the two populations display no differences in haplotype sharing patterns. In each of the three breeds analyzed, the populations from both countries largely show strong levels of similarity, both in phylogenetic relationship and ancestral breed hybridization yet two breeds, the Cane Corso and Italian Greyhound, show divergent genetic histories based on geographic barriers.

### 3.6 Timing of breed formation

We used the equation described by Parker et al. (Parker et al., 2017), to calculate the number of the years since shared genetic history was observed between breeds, based on the amount of haplotype sharing across breeds. A shared genetic history implies that the two breeds in question were either (1) the same population that diverged into two distinct populations or (2) two populations that showed substantial admixture. Therefore, the calculated time reflects the number of years prior to the date at which the genetic material was collected that the two breeds were last merged. The Italian breed samples utilized in this study were predominantly collected in 2016, so all admixture years were considered relative to that date. Most of the time points calculated with regard to the Italian breeds indicate admixture or divergence >100 years ago. Only eight breed pairs show admixture within 100 years of sample collection (Table S1). The Neapolitan Mastiffs from Italy and the United States have a negative value for years since admixture, indicating that they are of sufficient genetic similarity to be considered the same population. The same is true of Italian Greyhound populations from Italy and the United States despite the finding of different admixture components.

By comparison, the Neapolitan Mastiff and Cane Corso have a much shorter and more intertwined history and were recognized by the AKC in 2004 and 2010, respectively. Based on our haplotype analysis, the American Cane Corsos diverged from the Neapolitan Mastiff between 65 and 69 years ago, while the Italian population split 80–85 years ago. Written breed history argues that there was a strong effort to rescue what remained of the European Neapolitan Mastiff after World War II through implementation of a strict breeding program (Club 2012). Our data demonstrate that the modern Neapolitan Mastiff is genetically the same breed regardless of geography. American and Italian populations of Cane Corsos suggest a divergence of approximately 93 years ago and that this breed also underwent a restructuring after World War II. This divergence may reflect the presence of Cane Corsos in America prior to the breed resurrection or the effect of a migration bottleneck with distinct lineages contributing differentially to the two breed populations. Analysis of the Cirneco dell'Etna and Pharaoh Hound indicate haplotype sharing divergence of 65 years ago (1950), corresponding with the recognition of the breeds by the FCI. These results support the hypothesis that most breed formation occurred recently, within the last 200 years. But it also shows, importantly, that the genetic foundations of these breeds were laid in the more distant past.

## 4 DISCUSSION

### 4.1 Italian dog breeds

While most modern dog breeds have been developed in the past 200 years (Club, 2006, 2017; Lindblad‐Toh et al., 2005; Parker et al., 2017), some populations have taken recognizable forms, suited to distinct tasks, for much longer. Modern Italy stakes claim to a minimum of 23 dog varieties, of which 13 are internationally recognized, and 10 are known locally (Table 1). These breeds can be broadly classified into seven phenotypic categories: scent hound, flock guardian, mastiff, and sighthound, as were described by historical Roman authors (Xenophon; Columella, 1954), as well as the more modern hunting, herding, and companion breeds.

We have combined whole‐genome SNP data from 263 dogs representing 23 closed dog populations from Italy, seven Apennine wolves, and 161 purebred dog populations, and used multiple genetic methods to characterize the modes by which geographically distinct breeds are formed. Simultaneous consideration of each of five methodologies reveals a series of genetic profiles that both validate and expand historical records of breed creation, including divergence from a common population, regional stock influenced by foreign genetics, and globally isolated selection of local stock.

The purposes for which humans have directed the development of dog breeds are varied, yet remain predictable across cultures. The natural canine attributes of visual and olfactory acuity (Chen, Irwin, & Zhang, 2012; Tacher et al., 2005), speed and endurance (Huson et al., 2012; Kemp, Bachus, Nairn, & Carrier, 2005; Pasi & Carrier, 2003), guardianship, predatory nature (Akkad et al., 2015; Starling, Branson, Thomson, & McGreevy, 2013; Sundman, Johnsson, Wright, & Jensen, 2016), and their seemingly innate companionability with humans (Cagan & Blass, 2016; Fadel et al., 2016; Gacsi, McGreevy, Kara, & Miklosi, 2009; vonHoldt et al., 2017; Jakovcevic, Elgier, Mustaca, & Bentosela, 2010; van der Waaij, Wilsson, & Strandberg, 2008) have been exploited for thousands of years. However, in the pursuit of a distinct lineage, selection of breeding animals will unavoidably rely on a small source pool (Alam et al., 2012; Calboli, Sampson, Fretwell, & Balding, 2008; Kumpulainen et al., 2017; Pfahler & Distl, 2015; Streitberger et al., 2012; Wijnrocx et al., 2016). As such, dog breeds which perform similar tasks are frequently more closely related to each other than to breeds with different occupations, allowing for the visualization of phylogenetic breed clades sometimes segregating by broad behavior patterns (Parker et al., 2017; Vaysse, et al. 2011; von Holdt et al., 2010; Parker et al., 2004; and Figure 2). Within each of those application‐based clades, individual breeds may diverge by specialized skill, appearance, or environmental adaptation. We sought to match methodologies to modern breeds, looking for breed formation commonalities in subsets of phenotypes.

An example of breed development through divergence from a common genetic pool is the Small Spitz breeds. Phylogenetically, the Small Spitz clade (Figure 2) includes the Pomeranian, American Eskimo, and Volpino Italiano (Figure 1p) breeds. These breeds share a striking physical similarity, including small pointed muzzles, pricked triangular ears, profuse fur, and a plumed tail curled over the back. The Volpino Italiano shows genetic similarity to the Pomeranian. Haplotype sharing metrics suggest 1876 as the most recent instance of shared genetic history between the breeds. The formal recognition of the Pomeranian as a distinct breed in 1873 may have initiated the split between the populations. The Volpino Italiano regained popularity in 1968, after having fallen out of favor in the early 1900s (Peterson 2016). The rebuilding of the breed may have been aided by the classification of small spitz‐type breeds, initiated by the prior registration of Pomeranians, effectively concentrating the remaining Volpino Italianos through exclusion from other breed registries.

Breed development via geographic proximity and constraint is also well exemplified by consideration of Mediterranean sighthound breeds. Specifically, the Cirneco dell'Etna, a medium‐sized sighthound, is believed to have developed on the island of Sicily. The present genetic analyses place the Pharaoh Hound from the island of Malta and the Ibizan Hound from the island of Ibiza as the closest genetic relatives to the Cirneco dell'Etna. Haplotype sharing between the Cirneco dell'Etna and the Pharaoh Hound dates to 1950, reflective of FCI breed recognition of the breeds in 1956 and 1963, respectively. Conversely, the Levriero Meridionale from Southern Italy shows no heritage with the Cirneco dell'Etna and its close relatives, the Pharaoh and Ibizan hounds, but rather the Sloughi and Azawakh breeds originally from Northern Africa.

An additional example of genetic data superseding historical lore is that of the Segugio Italiano breed which, since 1989, has been classified as two separate breeds, the Pelo Raso (smooth haired) (Figure 1j) and Pelo Forte (rough haired) (Figure 1k) (Pallotti et al., 2017). Early writings by the Greek historian Arrian (c. 92‐175 AD) describe “Segusian hounds” as “shaggy” (Arrian, 1831), suggesting that the original breed may have more closely resembled the Pelo Forte of today. The genetic distance phylogeny and structure predictions presented here failed to reproducibly distinguish the two Segugio populations, implying that at the genetic level, they are the same breed. A similar relationship pattern was previously noted between the Tervuren and Groenendael varieties of Belgian Sheepdog, classified as separate breeds in the United States since 1959 (von Holdt et al., 2010; Parker et al., 2004, 2017). Considering both examples, then, it is not surprisingly that recent separation of breeding stock based on phenotype is inconsequential and does not override the genetic patterns laid out by a breed history.

### 4.2 Italian herding breeds and the German shepherd dog

Herding behavior is suggested to have arisen from multiple geographic and genetic backgrounds (Arnott, et al. 2015; Storteig Horn, 2017; Parker et al., 2017), forming separate clades termed Continental, UK Rural, Hungarian, and New World in recent analyses. The phylogenetic placement of the Italian herding breeds in the New World clade was therefore not necessarily predictable, as one would expect that each breed would be assigned by geographic location. The expanded dataset analyzed here not only placed them firmly in the New World clade, but in doing so resolved our understanding of the German Shepherd Dog's contribution to many clades of diverse types such as the Black Russian Terrier and Swedish Elkhound (Li et al., 2008), Portuguese Water Dog (von Holdt et al., 2010), Poodles (Fregel et al., 2015), and the Mexican and Peruvian hairless breeds, Berger Picard (Frantz et al., 2016; Parker et al., 2017).

The German Shepherd Dog displays significant haplotype sharing with several Mediterranean and other European breeds dated between 1859 and 1867, well before the establishment of the German Shepherd Dog as a unique breed in 1899. These estimated dates likely reflect the influence of a pervasive common livestock dog in continental Europe from which the German Shepherd Dog was formed (Caius, 1576; Pennant, 1793; Elite 2015). Haplotypes recognized in the modern German Shepherd Dog were then passed to the North American breeds during their own developmental period. This European agrarian signature is most readily visible in Figure 3a. The addition of the Italian breeds places an ancestor of the German Shepherd Dog as the connection between the larger population of herding breeds and the American breeds.

The six Italian herding breeds now cluster paraphyletic to the German Shepherd Dog, along with the previously mentioned Berger Picard, Chinook, Xoloitzcuintli, and Peruvian Inca Orchid. This result is striking given the differences in morphology and behavior between the breeds. Yet, the results make genetic sense. There were eight predicted admixture events in the New World clade based on the TREEMIX analysis, with two of them occurring in the direction from the German Shepherd Dog or Lupo Italiano to the Central American hairless breeds, while five introgression events indicate admixture of the German Shepherd Dog and the European herding breeds. The recurring pattern of a common genetic link to the German Shepherd Dog suggests a breed history that centers around a broadly distributed European herding dog stock, one that has given rise to the German Shepherd Dog, the Italian herding breeds, and the French Berger Picard.

### 4.3 Flock guardian breeds develop along lines of transhumance

The flock guardian breeds of Italy present a surprisingly accurate reflection of human industry. Management of domestic sheep and goats has been a key need since early human occupation, with skeletal remains of domestic caprines and dogs found in the Neolithic settlements of Sicily (Debono Spiteri et al., 2016; Leighton, 1999). The importance of the sheep industry in Italy continued through the Roman era (Canfield, 1853; Lambert 2014). Due to the topography and climate of the region, transhumance, the practice of moving sheep flocks and the dogs that protected them from mountainous pastures in summer to sheltered lowland pastures during winter, has long been employed. The standard routes, termed “tratturi,” between summer and winter pastures were etched into the landscape (Higgs, 1976; Pounds, 1990).

**Figure 4:**
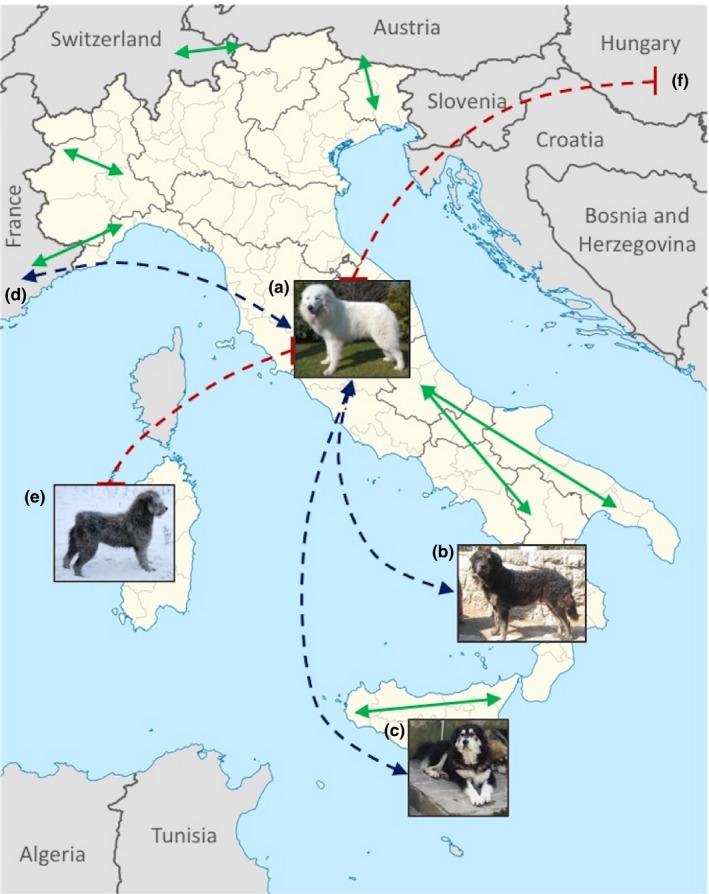
The agricultural practice of transhumance moves livestock along seasonal routes (green arrows). Haplotype sharing of flock guardian breeds (blue dashed arrows) follow these same routes, centering around the Maremma's Sheepdog (a), and including the Mannara's Dog (b), Pastore della Sila (c), and Great Pyrenees (d). The flock guardians of Sardinia, Fonni's Dog (e), and Hungary, Kuvasz and Komondor (f) do not share haplotypes with the mainland Italian flock guardian breeds (red dashed lines)

The data presented in this study link the four flock guardian breeds from peninsular Italy for the first time. Interestingly, while the Maremma Sheepdog displays genetic evidence of breed relationship to the remaining mainland flock guardians, the Mannara's Dog, Mastino Abruzzese, and Pastore della Sila do not show substantial genetic ties to each other. This pattern is distinct from that observed in the German Shepherd Dog, where 12 of the 19 breeds with significant haplotype sharing to the German Shepherd Dog also share with at least one other related breed. This suggests that the Maremma Sheepdog is reflective of the original flock guardian of the region and that, perhaps through the process of transhumance, seeded the distal pastoral flock guardian populations (Figure 4). In fact, the Mastino Abruzzese and the Maremma Sheepdog were historically considered separate breeds but, due to the proximity of ranges and migration of sheep flocks, were unified as a single breed, named Pastore Maremmano‐Abruzzese, in 1958 (Maremma Sheepdog Club of America 2017). Two samples were provided to us under the “Mastino Abruzzese” breed listing and analyzed in a previous publication (Parker et al., 2017). Given the recent time period during which they were received and the results of the expanded Italian breed analyses, we propose that they may represent the more modern Pastore Maremmano‐Abruzzese breed.

While the previous comparison demonstrates genetic relationships between dogs involved in transhumance, the same is lacking in dogs of similar occupation and physical phenotypes for which there is no evidence of seasonal migration. Specifically, haplotype sharing for other European flock guardian breeds demonstrates shared genetic signatures between the Great Pyrenees of France and Spain and the Italian Maremma Shepherd, but no genetic connection between the Hungarian Komondor and Kuvasz breeds to the Italian flock guardian breeds. Similarly, the Fonni's Dog, a flock guardian from the island of Sardinia, does not show a genetic relationship to the mainland flock guardian breeds. Transhumance was therefore a key force for breed formation along the mainland Italian peninsula, Alps, and Pyrenees mountains of France and Spain. However, in Hungary and Sardinia, in the absence of transhumance (Pounds, 1990), breed formation is more likely to occur by convergent selection, as demonstrated with the guardian breeds of the regions.

### 4.4 Summary

Human‐driven selection has yielded hundreds of dog breed variants. Utilizing 23 closed dog populations from a distinct geographic location, in‐depth demographic analyses relative to a large cohort of established dog breed populations reveals three modes through which selection has been applied: divergence from a large collection of phenotypically similar individuals; independently through repeated selection for a particular purpose without influence of introgression from other similar breeds; and inclusion of foreign breeding stock with desired traits. The present sampling of Italian breeds allows for the analysis of populations passing throughout multiple breed development stages.

The impact of human advancement on breed distribution and development is readily visible in relation to the agricultural practice of transhumance and the spread of livestock guardian breeds along the resultant routes. We have further elucidated a theory for a common agrarian shepherd dog type, regional to Central Europe, which likely existed prior to modern breed differentiation. The genetic signatures of this population can be recognized as having contributed to many regional breeds of European herding dogs, including the well‐known German Shepherd Dog, and the multiple Italian herding breeds adapted to working in the pastoral regions of Northern Italy. The analysis of additional regional dog breeds will expand our understanding of the impacts and routes of artificial selection, revealing ever more interplay between human and canine coevolution.

## CONFLICT OF INTEREST

The authors have no conflict of interests to declare.

## AUTHOR CONTRIBUTIONS

A.T. and D.L.D. contributed equally to this manuscript, through conceptualization, analyses, interpretation, and writing, and are to be considered co‐first authors. S.F., S.M., D.B., and R.C. provided resources and editing. M.P. and H.G.P. provided conceptualization and editing. L. L. and R. C. provided resources. G.P. provided departmental supervision. A.C.H. and A.N.H. provided sample management and processing. E.A.O. and P.C. provided conceptualization, funding acquisition, project administration and supervision, and editing.
