# Supplementary figures and images for: Studies of modern Italian dog populations reveal multiple patterns for domestic breed evolution
Source: Ecol Evol. 2018 Feb 14;8(5):2911–25. doi: 10.1002/ece3.3842 (PMC5838073; doi:10.1002/ece3.3842)

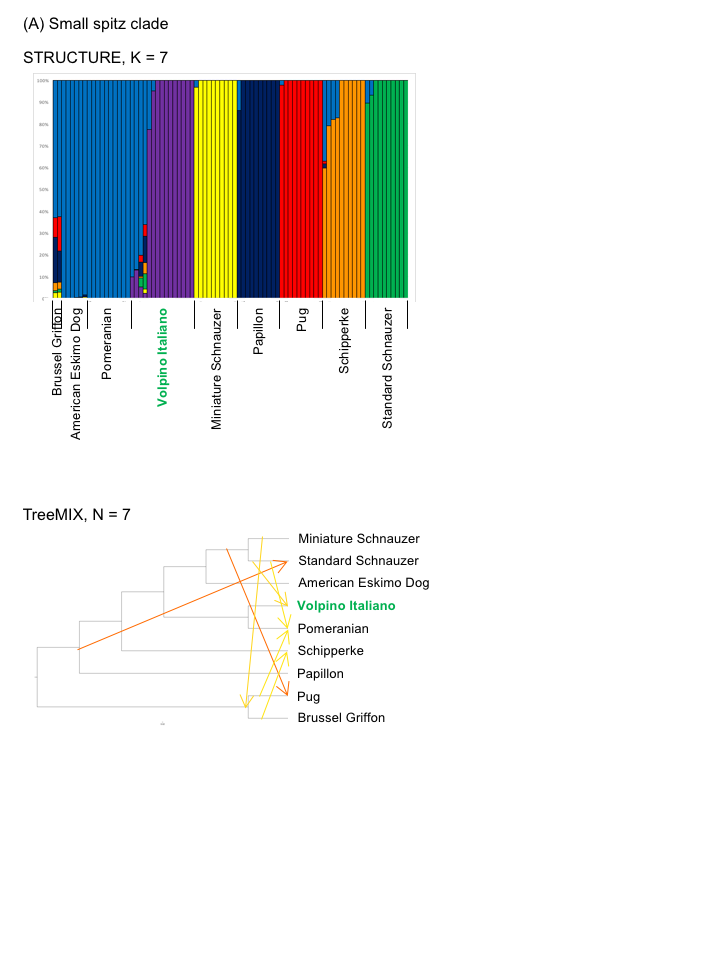

Supplement: Supplementary file 1 [file ECE3-8-2911-s001.tiff]

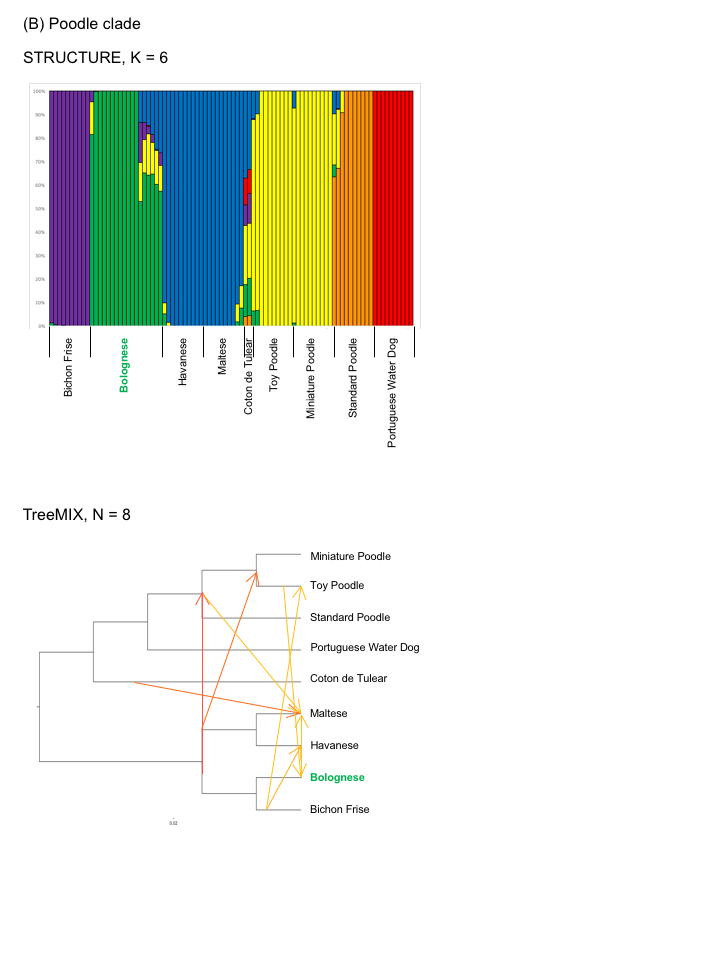

Supplement: Supplementary file 2 [file ECE3-8-2911-s002.tiff]

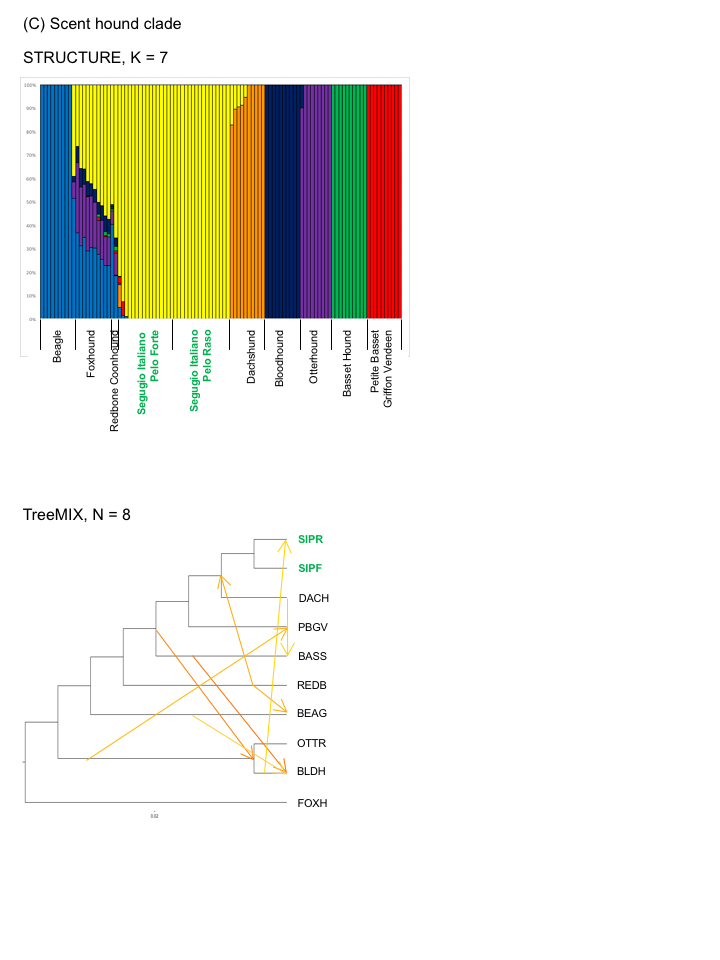

Supplement: Supplementary file 3 [file ECE3-8-2911-s003.tiff]

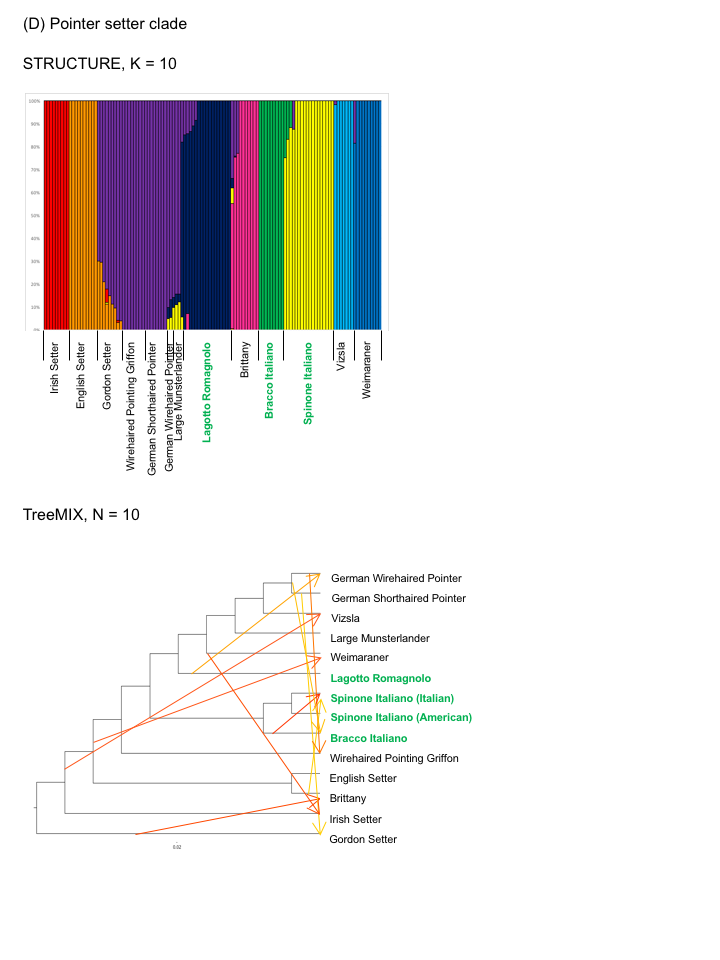

Supplement: Supplementary file 4 [file ECE3-8-2911-s004.tiff]

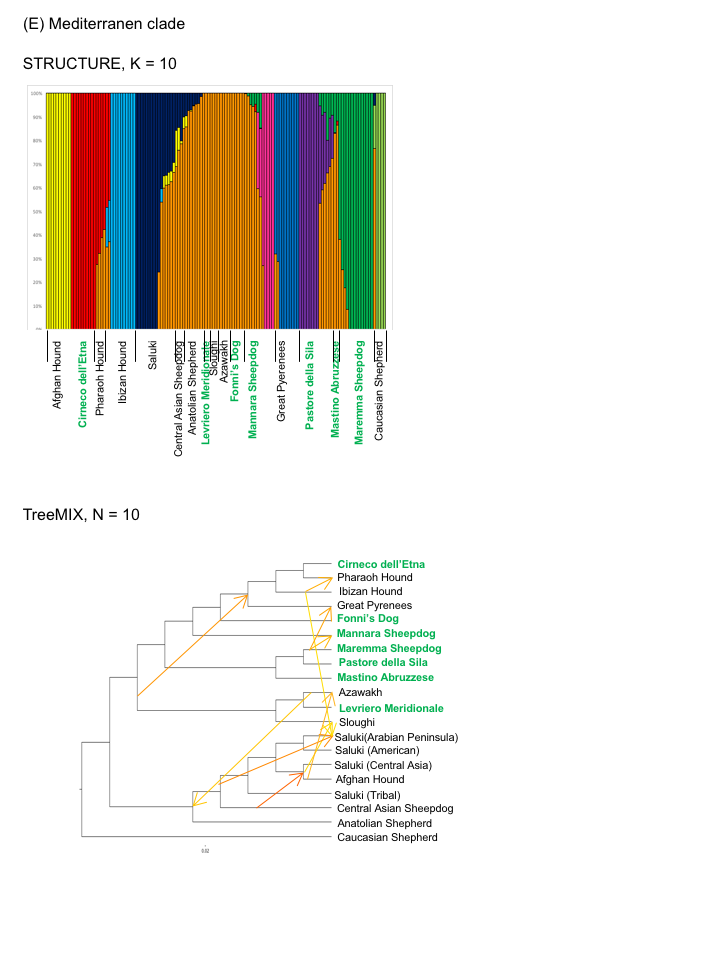

Supplement: Supplementary file 5 [file ECE3-8-2911-s005.tiff]

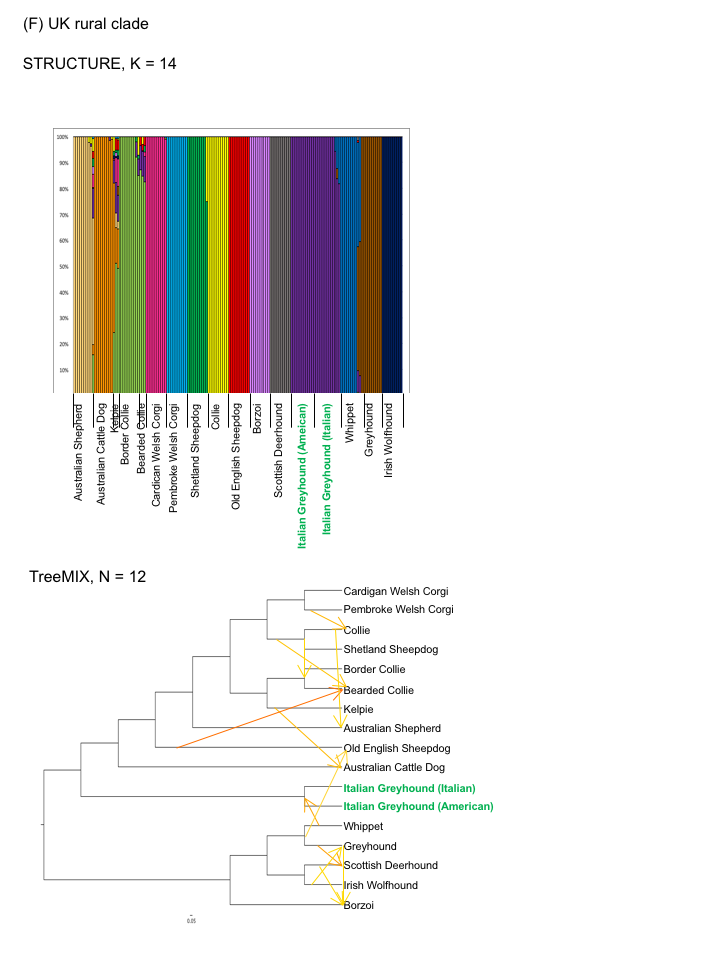

Supplement: Supplementary file 6 [file ECE3-8-2911-s006.tiff]
