# Supplementary material for: Studies of modern Italian dog populations reveal multiple patterns for domestic breed evolution
Source: Ecol Evol. 2018 Feb 14;8(5):2911–25. doi: 10.1002/ece3.3842 (PMC5838073; doi:10.1002/ece3.3842)
Supplement: Supplementary file 7 [file ECE3-8-2911-s007.docx]

**Supplemental Table 1:** Instances of significant identity-by-descent haplotype sharing with each of the Italian dog populations.

| **Breed 1** | **Breed 2** | **Haplotype Sharing (bp)^a^** | **Admixture Years^b^** |
| --- | --- | --- | --- |
| Bergamasco Shepherd | German Shepherd Dog | 27,138,362 | 145.68 |
|  | Lupino del Gigante | 17,193,114 | 151.85 |
|  | Briard | 16,487,426 | 152.29 |
|  | Pastore della Lessinia e del Lagorai | 16,034,057 | 152.57 |
|  | Cane Paratore | 15,050,300 | 153.18 |
|  | Lupo Italiano | 13,139,089 | 154.36 |
|  | Collie | 12,571,331 | 154.71 |
|  | Berger Picard | 12,464,003 | 154.78 |
|  | Chinook | 11,462,525 | 155.40 |
|  | Pastore d’Oropa | 10,859,649 | 155.77 |
|  | Shetland Sheepdog | 10,075,144 | 156.26 |
|  | Bernese Mountain Dog | 9,587,046 | 156.56 |
| Bolognese | Bichon Frise | 48,775,338 | 132.27 |
|  | Maltese | 31,040,322 | 143.26 |
|  | Havanese | 22,978,422 | 148.26 |
|  | Miniature Poodle | 18,695,854 | 150.92 |
|  | Toy Poodle | 17,499,910 | 151.66 |
|  | Pug | 10,181,680 | 156.20 |
| Bracco Italiano | Spinone Italiano | 47,008,274 | 133.37 |
|  | German Shorthaired Pointer | 13,215,965 | 154.31 |
|  | English Setter | 11,788,810 | 155.20 |
|  | Wirehaired Pointing Griffon | 11,694,573 | 155.26 |
|  | Vizsla | 11,136,141 | 155.60 |
|  | German Wirehaired Pointer | 11,096,019 | 155.63 |
|  | Brittany | 10,003,250 | 156.31 |
| Cane Corso (US) | Neapolitan Mastiff (Italy) | 156,885,272 | 65.25 |
|  | Neapolitan Mastiff (US) | 150,362,353 | 69.29 |
|  | Cane Corso (Italy) | 112,208,668 | 92.95 |
|  | Boxer | 58,805,030 | 126.05 |
|  | Bullmastiff | 40,766,438 | 137.23 |
|  | Mastiff | 29,228,160 | 144.39 |
|  | Boerboel | 19,319,509 | 150.53 |
|  | Rottweiler | 16,724,158 | 152.14 |
|  | Dogue de Bordeaux | 15,011,022 | 153.20 |
|  | Bulldog | 11,670,780 | 155.27 |
|  | St Bernard | 11,538,514 | 155.35 |
| Cane Corso (Italy) | Neapolitan Mastiff (Italy) | 133,180,436 | 79.94 |
|  | Neapolitan Mastiff (US) | 124,325,648 | 85.43 |
|  | Cane Corso (US) | 112,208,668 | 92.95 |
|  | Boxer | 75,814,140 | 115.51 |
|  | Bullmastiff | 38,811,286 | 138.45 |
|  | Mastiff | 28,890,929 | 144.60 |
|  | Dogue de Bordeaux | 19,992,346 | 150.11 |
|  | Boerboel | 19,984,729 | 150.12 |
|  | Bulldog | 16,609,874 | 152.21 |
|  | St Bernard | 14,593,977 | 153.46 |
| Cirneco dell’Etna | Pharaoh Hound | 156,471,556 | 65.51 |
|  | Ibizan Hound | 47,368,562 | 133.14 |
| Cane Paratore | German Shepherd Dog | 67,689,477 | 120.54 |
|  | Lupino del Gigante | 34,801,792 | 140.93 |
|  | Berger Picard | 32,797,784 | 142.17 |
|  | Lupo Italiano | 31,773,751 | 142.81 |
|  | Pastore della Lessinia e del Lagorai | 27,779,726 | 145.29 |
|  | Chinook | 15,423,705 | 152.95 |
|  | Bergamasco Shepherd | 15,050,300 | 153.18 |
|  | Xoloitzcuintle | 14,915,146 | 153.26 |
|  | Belgian Malinois | 13,907,879 | 153.89 |
|  | Pastore d’Oropa | 11,821,454 | 155.18 |
|  | Boxer | 11,595,004 | 155.32 |
| Fonni’s Dog | German Shepherd Dog | 14,213,369 | 153.70 |
| Italian Greyhound (US) | Italian Greyhound (Italy) | 265,191,294 | *0* |
|  | Whippet | 25,881,592 | 146.46 |
|  | Greyhound | 14,984,034 | 153.22 |
|  | Scottish Deerhound | 11,221,646 | 155.55 |
|  | Toy Manchester Terrier | 10,398,130 | 156.06 |
| Italian Greyhound (Italy) | Italian Greyhound (US) | 265,191,294 | *0* |
|  | Whippet | 25,790,773 | 146.52 |
|  | Greyhound | 17,021,110 | 151.96 |
|  | Scottish Deerhound | 13,021,386 | 154.43 |
| Lagotto Romagnolo | English Setter | 17,368,800 | 151.74 |
|  | Wirehaired Pointing Griffon | 9,851,734 | 156.40 |
|  | German Wirehaired Pointer | 9,572,134 | 156.57 |
| Levriero Meridionale | Sloughi | 43,424,896 | 135.59 |
|  | Azawakh | 25,976,838 | 146.40 |
| Lupino del Gigante | German Shepherd Dog | 71,723,422 | 118.04 |
|  | Lupo Italiano | 34,808,174 | 140.93 |
|  | Cane Paratore | 34,801,792 | 140.93 |
|  | Berger Picard | 32,893,914 | 142.12 |
|  | Pastore della Lessinia e del Lagorai | 29,339,947 | 144.32 |
|  | Chinook | 22,505,077 | 148.56 |
|  | Bergamasco Shepherd | 17,193,114 | 151.85 |
|  | Pastore d’Oropa | 16,044,728 | 152.56 |
|  | Belgian Sheepdog | 15,301,850 | 153.02 |
|  | Belgian Tervuren | 14,993,743 | 153.21 |
|  | Belgian Malinois | 14,177,835 | 153.72 |
|  | Xoloitzcuintle | 12,178,144 | 154.96 |
|  | Collie | 11,335,572 | 155.48 |
|  | Leonberger | 9,924,263 | 156.35 |
| Lupo Italiano | German Shepherd Dog | 87,699,657 | 108.14 |
|  | Lupino del Gigante | 34,808,174 | 140.93 |
|  | Berger Picard | 32,776,942 | 142.19 |
|  | Cane Paratore | 31,773,751 | 142.81 |
|  | Pastore della Lessinia e del Lagorai | 29,158,139 | 144.43 |
|  | Chinook | 19,975,816 | 150.12 |
|  | Pastore d’Oropa | 16,132,135 | 152.51 |
|  | Bergamasco Shepherd | 13,139,089 | 154.36 |
|  | Xoloitzcuintle | 13,119,334 | 154.37 |
|  | Peruvian Inca Orchid | 9,734,744 | 156.47 |
| Mannara’s Dog | Maremma Sheepdog | 13,939,808 | 153.87 |
|  | German Shepherd Dog | 10,030,540 | 156.29 |
| Maremma Sheepdog | Pastore della Sila | 18,951,808 | 150.76 |
|  | Great Pyrenees | 14,714,110 | 153.39 |
|  | Mannara’s Dog | 13,939,808 | 153.87 |
|  | Mastino Abruzzese | 13,814,472 | 153.94 |
| Mastino Abruzzese | Maremma Sheepdog | 13,814,472 | 153.94 |
|  | German Shepherd Dog | 10,194,214 | 156.19 |
| Neapolitan Mastiff (US) | Neapolitan Mastiff (Italy) | 465,111,124 | *0* |
|  | Cane Corso (US) | 150,362,353 | 69.29 |
|  | Cane Corso (Italy) | 124,325,648 | 85.43 |
| Neapolitan Mastiff (Italy) | Neapolitan Mastiff (US) | 465,111,124 | *0* |
|  | Cane Corso (US) | 156,885,272 | 65.25 |
|  | Cane Corso (Italy) | 133,180,436 | 79.94 |
| Pastore della Lessinia e del | German Shepherd Dog | 53,461,528 | 129.36 |
| Lagorai | Lupino del Gigante | 29,339,947 | 144.32 |
|  | Lupo Italiano | 29,158,139 | 144.43 |
|  | Belgian Sheepdog | 28,240,057 | 145.00 |
|  | Cane Paratore | 27,779,726 | 145.29 |
|  | Collie | 26,882,214 | 145.84 |
|  | Berger Picard | 23,040,880 | 148.22 |
|  | Belgian Tervuren | 22,519,315 | 148.55 |
|  | Chinook | 19,636,516 | 150.33 |
|  | Shetland Sheepdog | 18,469,946 | 151.06 |
|  | Bergamasco Shepherd | 16,034,057 | 152.57 |
|  | Belgian Malinois | 14,654,568 | 153.42 |
|  | Pastore d’Oropa | 11,993,182 | 155.07 |
| Pastore della Sila | Maremma Sheepdog | 18,951,808 | 150.76 |
|  | German Shepherd Dog | 15,378,975 | 152.97 |
|  | Rottweiler | 11,207,564 | 155.56 |
| Pastore d’Oropa | German Shepherd Dog | 31,567,625 | 142.94 |
|  | Collie | 16,464,082 | 152.30 |
|  | Lupo Italiano | 16,132,135 | 152.51 |
|  | Lupino del Gigante | 16,044,728 | 152.56 |
|  | Chinook | 15,999,428 | 152.59 |
|  | Berger Picard | 14,026,042 | 153.81 |
|  | Pastore della Lessinia e del Lagorai | 11,993,182 | 155.07 |
|  | Cane Paratore | 11,821,454 | 155.18 |
|  | Bergamasco Shepherd | 10,859,649 | 155.77 |
| Segugio Italiano Pelo Forte | Segugio Italiano Pelo Raso | 57,370,060 | 126.94 |
| Segugio Italiano Pelo Raso | Segugio Italiano Pelo Forte | 57,370,060 | 126.94 |
|  | Bloodhound | 10,541,864 | 155.97 |
|  | Basset Hound | 9,844,092 | 156.40 |
|  | Foxhound | 9,501,742 | 156.62 |
|  | Otterhound | 9,279,222 | 156.75 |
| Spinone Italiano | Bracco Italiano | 47,008,274 | 133.37 |
|  | English Setter | 15,748,798 | 152.74 |
|  | Wirehaired Pointing Griffon | 14,693,518 | 153.40 |
|  | German Shorthaired Pointer | 12,651,350 | 154.66 |
|  | Gordon Setter | 10,313,656 | 156.11 |
|  | Large Munsterlander | 10,270,502 | 156.14 |
|  | Brittany | 9,758,386 | 156.46 |
|  | Irish Setter | 9,596,070 | 156.56 |
| Volpino Italiano | Pomeranian | 36,957,865 | 139.60 |
|  | Standard Schnauzer | 15,246,342 | 153.06 |
|  | Miniature Schnauzer | 14,604,524 | 153.45 |
|  | American Eskimo Dog | 14,235,331 | 153.68 |
|  | Keeshond | 13,935,570 | 153.87 |
|  | Brussels Griffon | 10,537,711 | 155.97 |
|  | Icelandic Sheepdog | 10,420,440 | 156.05 |
|  | Pug | 9,325,924 | 156.73 |
| Apennine Wolf | Wolf | 17,187,281 | 151.85 |

^a^ Haplotype sharing is considered to be significant when over 9,257,455 bp, top 5% of sharing events.

^b^ The number of years, prior to 2016, in which the two breeds are calculated to have had a shared genetic history, based on the length of haplotype sharing.
